# Evaluating the Impact of Pediatric Digital Mental Health Care on Caregiver Burnout and Absenteeism: Longitudinal Observational Study

**DOI:** 10.2196/67149

**Published:** 2025-06-27

**Authors:** Darian Lawrence-Sidebottom, Kelsey McAlister, Aislinn Brenna Beam, Rachael Guerra, Amit Parikh, Monika Roots, Donna McCutchen, Landry Goodgame Huffman, Jennifer Huberty

**Affiliations:** 1 Bend Health, Inc, 321 East Washington Ave #200, Madison, WI, 53703, United States, 1 8005160975; 2 Fit Minded, Inc, Phoenix, AZ, United States

**Keywords:** digital mental health intervention, mental health, mental illnesses, mental disorders, child behavioral health, child behavior, workplace outcomes, pediatrics, children, youth, adolescents, adolescence, teenagers, digital, digital health, digital technology, digital interventions

## Abstract

**Background:**

Caregivers of children with mental health challenges are at heightened risk for burnout and absenteeism. This strain affects both their well-being and work performance, contributing to widespread workplace issues. Digital mental health interventions (DMHIs) are increasingly used to support pediatric mental health, but their impact on caregiver outcomes remains underexplored.

**Objective:**

This study aimed to explore the associations between caregiver burnout, absenteeism (ie, missing work), comorbid symptoms, and child mental health problems, and to assess whether caregiver burnout and absenteeism improved as their child participated in a pediatric DMHI.

**Methods:**

This retrospective study included 6506 caregivers whose children (aged 1‐17 years) received care from Bend Health, Inc, a pediatric DMHI providing digital-based therapy and coaching, digital content, and caregiver support. Caregiver burnout, absenteeism, comorbid symptoms, and child mental health symptoms were measured by monthly assessments. Cumulative link models were used to assess the associations of between child symptoms and caregiver outcomes and to assess changes in caregiver outcomes over the course of the DMHI. Analyses of baseline associations included the full sample (n=6506), while analyses of pre-post changes in caregiver outcomes were conducted in caregivers with elevated burnout (n=2121) and absenteeism (n=1327) who had an assessment after starting care.

**Results:**

At baseline, 45.96% (2990/6506) of caregivers reported elevated burnout and 28.96% (1884/6506) reported elevated absenteeism. More severe burnout was associated with having a child with elevated symptoms of any type (all *P*<.01). More severe absenteeism was significantly associated with having a child with elevated symptoms of depression (*z*=3.33; *P*<.001), anxiety (*z*=3.96; *P*<.001), inattention (*z*=2.48; *P*=.013), and hyperactivity (*z*=2.12; *P*=.03). Burnout decreased for 68.64% (1456/2121) and absenteeism decreased for 87.26% (1158/ 1327). Greater months in care was associated with less severe caregiver burnout (*z*=−5.48; *P*<.001) and absenteeism (*z*=−6.74; *P*<.001).

**Conclusions:**

DMHIs for children may reduce caregiver burnout and absenteeism. These findings emphasize the value of employers offering pediatric DMHIs as part of employee benefits, potentially enhancing workplace outcomes.

## Introduction

Children and adolescents in the United States aged 3-17 years are experiencing record levels of mental and behavioral health issues such as anxiety, depression, and attention-deficit/hyperactivity disorder (ADHD), which can contribute to increased stress, fatigue, sleep disturbances, and overall strain on family well-being [1,2]. Alongside the decline of pediatric mental health in recent years, fewer caregivers have reported that they are coping with the demands of parenting, citing increased emotional distress, worry, feelings of helplessness, and family strain [2-5]. As a response to the crisis in caregiver mental health and well-being, the US Surgeon General issued a 2024 advisory calling for greater support for parent and caregiver mental health to improve family well-being [4].

There is substantial evidence that caregivers of children with mental health challenges are particularly vulnerable to poor mental health outcomes, as they work to meet their children’s complex needs [6,7]. Caregivers whose children experience emotional and behavioral problems are under additional strain, as they frequently experience elevated parental stress, fatigue, and poor sleep [6-11]. This degradation of caregiver well-being ultimately spills into different areas of life [12], including both family and professional responsibilities.

In a survey of parents who were concerned about their children’s mental health, 48% said that their concerns impacted their performance at work in some way—including challenges such as frequent disruptions during the workday and difficulty concentrating on the job [13]. Caregivers may also have to miss work, given the need to take time off for their child’s medical appointments, as well as to tend to their own recuperation and care [13,14]. For example, 46% of caregivers of a child with ADHD reported reducing their weekly work hours, and 11% stopped working altogether after their child’s diagnosis [15]. Burnout and absenteeism impact both caregivers and employers, leading to reduced work performance, higher employee turnover, and significant productivity costs [16,17]. Thus, the issue of caregiver well-being is critical for society as a whole, including the health care system, individuals and families, and also businesses. Given evidence suggesting the deterioration of caregiver workplace outcomes associated with caring for a child with mental health challenges, there is a need for effective treatments for both child and caregiver well-being.

Digital mental health interventions (DMHIs) in academic and commercial settings have proven effective in treating common mental and behavioral health problems in children [18-22], offering accessible and cost-effective solutions compared with standard care [23,24]. Our previous research demonstrates that a commercially available DMHI can effectively improve mental health in children and adolescents [18,19]. Additionally, multiple systematic reviews highlight DMHIs as a highly promising solution for youth mental health [20,21]. More recently, a digital platform integrating both asynchronous and synchronous support successfully reduced anxiety and depression in a large sample of Australian youth [22]. Although these interventions primarily focus on the child, our previous findings suggest that caregivers may experience secondary benefits (eg, improvements in sleep and stress) when their children receive care through a pediatric DMHI [10]. However, the impact of pediatric DMHIs on caregiver burnout and absenteeism remains underexplored. This gap in knowledge is critical, as understanding caregiver well-being is essential to supporting their ability to manage both their child’s treatment and their own work and personal responsibilities effectively.

Using retrospective analyses, the purpose of this study was to (1) explore associations between caregiver burnout, absenteeism, comorbid symptoms, and child mental health problems among caregivers seeking mental health treatment for their children, and (2) assess whether caregiver burnout and absenteeism improved while their child participates in a pediatric DMHI.

## Methods

### Design and Participants

This study is part of a broader research effort examining the impact of a pediatric DMHI on child and caregiver outcomes. While we have published other analyses among youth and caregivers who participate in care with Bend Health [10,18,19,25], this is the first study to specifically assess the relationship between child mental health challenges and caregiver workplace outcomes (absenteeism and burnout). Caregivers of children (aged 1-17 years) participating in care with Bend Health, Inc, a collaborative care pediatric DMHI, were eligible for inclusion in this study if (1) they completed the caregiver assessments (burnout, absenteeism, sleep problems, and parental stress) at baseline (before the start of care), and (2) their child attended at least 1 session with a Bend Health practitioner (behavioral care manager [BCM], behavioral health coach [coach], or therapist) between January 1, 2023, and September 16, 2024. A total of 6508 caregivers were eligible for inclusion.

### Ethical Considerations

Caregivers provide informed consent during enrollment in care with Bend Health, Inc, for primary data collection required for regular participation in care and they also agree to the use of their data in further analyses. Caregivers agree to these terms on behalf of their children and themselves, and adolescents (aged 13-17 years) also assent on their own behalf. For the purposes of this study, only deidentified data were used. There was no compensation for participation in this study because the analysis was retrospective. Study procedures were approved by the Biomedical Research Alliance of New York (Study 23-12-034-1374; approved June 5, 2023). No portion of this manuscript used generative artificial intelligence for development or writing.

### Treatment

Bend Health, Inc, is a collaborative care DMHI, which delivers comprehensive mental health care to members (aged 1-17 years) and their caregivers. Care with Bend Health is described elsewhere in more detail [18,19]. Briefly, members can enroll via referral from their primary care provider, via insurance or employer benefits, or through direct-to-consumer channels. After enrollment in the web-based platform, a BCM is assigned to each member to coordinate care and communicate with external providers (eg, primary care providers), per the collaborative care model. The BCM assembles the care team, assigning a coach, and sometimes a therapist, based on coverage and each member’s unique needs, and then they continue to oversee each member’s care. Module-based care programs are assigned to each member to directly address a particular symptom domain (eg, anxiety or depression) and in an age-appropriate manner. In synchronous video-based sessions, the practitioners on a member’s care team deliver evidence-based care as aligned with the care plan. Members may attend multiple sessions monthly with their care team, and they may also receive care from a psychiatric provider if medication management is referred. While care with the DMHI targets the pediatric member, caregivers are closely involved in care and may receive mental health and behavior change tools to implement personally or in their interactions with their child or adolescent. Caregivers are required to attend sessions for safety reasons if their child is younger than 13 years.

### Measures

The member’s (ie, the child’s) demographic information, including the date of birth, sex at birth (male, female, or other), gender (male, female, transgender, nonbinary, or other), and race or ethnicity, is provided by caregivers at enrollment with Bend Health, Inc. The race or ethnicity options are specified in Textbox 1. Starting May 26, 2023, the options were expanded to be more inclusive of a diverse population, and multiple responses were allowed.

Textbox 1. **Race or ethnicity response options during enrollment, including options added to the demographic questions partway through the study period.**

Ethnicity response options

- American Indian or Alaska Native
- Asian
- Black or African American
- Hispanic or Latino
- Native Hawaiian or other Pacific Islander
- White
- Other (removed from the list of options starting May 26, 2023)
- Chinese (added to the list of options starting May 26, 2023)
- Vietnamese (added to the list of options starting May 26, 2023)
- Filipino (added to the list of options starting May 26, 2023)
- Korean (added to the list of options starting May 26, 2023)
- Japanese (added to the list of options starting May 26, 2023)
- Chamorro (added to the list of options starting May 26, 2023)
- Other Asian (added to the list of options starting May 26, 2023)
- Some other race or multi-racial (added to the list of options starting May 26, 2023)
- Mexican, Mexican Am, Chicano (added to the list of options starting May 26, 2023)
- Puerto Rican (added to the list of options starting May 26, 2023)
- Cuban (added to the list of options starting May 26, 2023)
- Another Hispanic, Latino, or Spanish origin (added to the list of options starting May 26, 2023)

As part of care at Bend, caregivers and adolescents (aged 13-17 years) complete mental health assessments during enrollment, and they also complete follow-up assessments every month during care to measure both caregiver and member symptoms. Caregivers complete assessments to measure caregiver burnout, absenteeism (missing work), sleep problems, and parental stress, as well as inattention, hyperactivity, and oppositional symptoms for their child or adolescent (caregiver report). Caregivers of children (aged 1-12 years) also complete assessments to measure their child’s anxiety, depressive symptoms, and sleep problems (caregiver report). Adolescents (aged 13-17 years) complete their own assessments to measure anxiety, depression, and sleep problems (self-report).

For all assessments except burnout, screener items are used to flag symptoms requiring further assessment. When responses to the screener items are flagged (indicating probable symptoms), the caregiver or the adolescent screens-in and completes a full validated assessment. In 2024, Bend Health’s method of screening-in to the validated assessments changed. From January 1, 2023, to January 22, 2023, caregivers and adolescents completed all screeners at each assessment, followed by validated assessments based on screener responses. From January 23, 2024, onward, if an assessment is screened-in, all following assessments for that symptom are automatically screened-in. From January 23, 2024, to August 9, 2024, screeners were not taken if past assessments were screened-in. From August 9, 2024, onward, screeners were taken in addition to validated assessments when past assessments were screened-in. Once all assessments are completed (after approximately 5-25 minutes), the caregiver and child or adolescent are shown a summary of their results, including an indication of whether any symptoms were mild to severe. The assessment results are also viewed by BCMs and the care team to monitor treatment progress and make adjustments to the care plan, as necessary.

#### Caregiver Symptoms

Caregiver symptoms were assessed using Bend Health’s regular symptom assessments, which measure a caregiver’s well-being while their child is in care. To assess caregivers’ work burnout, caregivers respond to a single question [16,26]: “Overall, based on your definition of burnout, how would you rate your level of burnout?” Caregivers respond using the following options: (1) “I enjoy my work. I have no symptoms of burnout” (no burnout), (2) “Occasionally I am under stress, and I don’t always have as much energy as I once did, but I don’t feel burned out” (low burnout), (3) “I am definitely burning out and have one or more symptoms of burnout such as physical and emotional exhaustion” (mild burnout), (4) “The symptoms of burnout that I’m experiencing won’t go away. I think about frustration at work a lot” (moderate burnout), and (5) “I feel completely burned out and often wonder if I can go on. I am at the point where I may need some changes or may need to seek some sort of help” (severe burnout). Studies have demonstrated that this single-time burnout measure is valid and reliable [26-28]. This item has demonstrated good concurrent validity with the emotional exhaustion subscale of the Maslach Burnout Inventory, supporting its reliability in assessing burnout [29].

To assess caregivers’ work absenteeism, defined as missing full or partial days of work, caregivers are asked the screening question: “During the past four (4) weeks, have you missed part of or an entire day of work? Have you had to come in early, go home late or work on your day off?” Responses are “Yes” or “No,” and caregivers are prompted to complete section B5 of the Health and Work Performance Questionnaire (HPQ) if they respond “Yes” [30]. B5 of the HPQ includes 5 items asking the number of days (range: 0-28) in the past 4 weeks that the caregiver (1) missed an entire day of work due to problems with their own physical or mental health, (2) missed an entire day of work for any other reason (including vacation), (3) missed part of a workday due to problems with their own physical or mental health, (4) missed part of a workday for any other reason (including vacation), and (5) came in early, went home late, or worked on a day off (extended workdays). The final item is not used to assess absenteeism and thus was not considered in this *study. Section* B5 of the HPQ has demonstrated good validity, showing strong correlations with objective measures of work performance and self-reported job performance [30].

To assess caregiver sleep problems, caregivers are asked to respond to the screener question: “During the past two (2) weeks, how much (or how often) have you had problems sleeping—that is, trouble falling asleep, staying asleep, or waking up too early?” Responses are on a 5-item Likert-type scale (0=Not at all, 4=Nearly every day). If the response is 2 or greater, the caregiver completes the Insomnia Severity Index (ISI), which includes 7 items about sleep difficulties [31]. Responses to each question are on a 5-item Likert-type scale (0=None, 5=Very severe). The ISI shows strong validity, correlating well with clinician-rated insomnia and sleep diary measures [31].

To assess parental stress, caregivers complete the Parental Stress Scale (PSS) [32], which queries a caregiver’s feelings about their caregiving responsibilities and their relationship with their child or children. First, they respond to the following 2 items from the PSS: “The major source of stress in my life is my child” and “Having a child leaves little time and flexibility in my life.” Best-fit responses are selected on a 5-item Likert-type scale (1=Strongly disagree, 5=Strongly agree). If the response to either question is 3 (undecided) or greater, the caregiver completes the remaining 16 items of the PSS. Some items on the PSS are framed as a negative caregiving experience (eg, “Having children has been a financial burden”), with greater responses indicating more severe parental stress. Other items are framed as a positive caregiving experience (eg, “I am happy in my role as a parent”), with greater responses indicating less severe parental stress (reverse items). The PSS shows good validity, correlating strongly with related measures of parental stress and well-being [32].

#### Child and Adolescent Mental Health Symptoms

All assessments of mental health for children (aged 1-12 years) are completed by the caregiver (caregiver report), including assessments of anxiety, depression, sleep problems, ADHD symptoms (inattention, hyperactivity, and opposition), and sleep problems. Adolescents (aged 13-17 years) complete their own assessments of anxiety, depression, and sleep problems (self-report), but their caregivers complete the assessment for ADHD, given the need for proxy report on these symptoms. For all assessments, the reporter (caregiver or self) responds to a few screening items to flag mental health symptoms. If mental health symptoms are flagged by these screeners, the reporter completes a full validated assessment to further quantify outcomes.

For child symptoms of anxiety, depression, and sleep problems, caregivers of children respond to screener items from the *Diagnostic and Statistical Manual of Mental Disorders* (Fifth Edition, Text Revision) (*DSM-V-TR*) Cross-Cutting Symptom Measure screeners [33]. If a response of 2 or greater is given to any screener, they complete the corresponding PROMIS (Patient-Reported Outcomes Measurement Information System) anxiety, depression, or sleep assessment [34]. Responses to the anxiety and depression PROMIS assessments are made using a 5-item Likert scale, with greater response values always indicating more severe or frequent symptoms (1=Never, 5=Almost always). Responses to the sleep PROMIS assessment are made using different 5-item Likert scales (depending on the question; eg, 1=Not at all, 5=Very much), with greater responses on some items indicating more severe or frequent symptoms and greater responses on other items indicating less severe or infrequent symptoms (reverse items).

For adolescent symptoms of anxiety, depression, and sleep problems, adolescents respond to items derived from the following respective screeners: Generalized Anxiety Disorder 2-item (anxiety) [35], Patient Health Questionnaire 2-item (depression) [36], and *DSM-V-TR* Cross-Cutting Symptom Measure screener for sleep (sleep problems) [33]. If there is an aggregate screener score of 2 or greater, they complete the corresponding validated assessment. For anxiety symptoms, adolescents complete the full Generalized Anxiety Disorder 7-item (GAD-7) [35]. For depressive symptoms, adolescents complete a version of the Patient Health Questionnaire 9-item that is modified for adolescents (PHQ-9A), excluding the item about suicidal ideation [36]. For sleep problems, adolescents complete the self-report version of the PROMIS sleep assessment [37], which includes the same items as the caregiver report version used for children.

For child and adolescent symptoms of ADHD, caregivers respond to 2 *DSM-V-TR* Cross-Cutting Symptom Measure items to screen for symptoms of inattention and hyperactivity (1 question) and opposition (1 question) [33]. If symptoms are flagged given responses to these screeners, caregivers complete all of or some of the subsets of the Swanson Nolan and Pelham Rating Scale version 4 (SNAP-IV) assessment [35], which includes 3 groups of questions measuring symptoms of inattention (items 1‐9), hyperactivity (items 10‐18), and opposition (items 19‐26). Responses to all items are on a Likert scale from 0 (Not at all) to 3 (Very much). From January 1, 2023, to March 31, 2024, a score of 1 or greater to the screener about inattention and hyperactivity prompted the completion of only the inattention and hyperactivity items, and a score of 1 or greater to the screener about opposition prompted the completion of only the opposition items [6]. Beginning April 1, 2024, caregivers complete all 26 questions of the SNAP-IV if their response to either screening question is 1 or greater. See the Multimedia Appendix 1 for further details on all measures used.

#### Outcome Calculations

Caregiver outcomes were calculated as follows. Using established criteria [27], elevated burnout was defined as a burnout score of 3 or more (mild to severe burnout). For absenteeism, reported partial days of work missed (items 3 and 4) were considered 0.5 days missed, so days of work missed was calculated as the sum of the number of full days missed (items 1 and 2) plus the sum of the number of partial days missed: item response 1 + item response 2+ (0.5 × (item response 3+ item response 4)). If the absenteeism assessment was screened-out, the number of days of work missed was 0. If the number of days of work missed exceeded 28, this value was replaced with 28. Given that the standard work week is five 8-hour days, severity of absenteeism was determined as follows: “No missed work” (screen-out or days of work missed is 0), “Missed less than one week of work” (days of work missed is 0.5-4.5), “Missed one to two weeks of work” (days of work missed is 5-9.5), “Missed two to three weeks of work” (days of work missed is 10-14.5), and “Missed three or greater weeks of work” (days of work missed is 15 or greater). Elevated absenteeism was considered missing work for 0.5 days or greater in the past 28 days. Caregiver sleep score was calculated by aggregating the responses to all 7 ISI items, for a total score of 0-28. Elevated sleep problems were considered moderate severity or severe insomnia symptoms, per the defined ranges of clinically significant sleep problems [31]. Parental stress was calculated by aggregating responses to the 18 items (with scores reversed for the reverse items), for a total score of 18-90. Elevated parental stress was considered a score of 42 or greater, given severity thresholds used by others [38,39].

Child and adolescent mental health outcomes were calculated as follows. Child anxiety and depressive symptom scores, as well as child and adolescent sleep scores, were calculated by aggregating the responses to the respective PROMIS assessments (with scores reversed for the reverse items) and then converting these total scores to *T*-scores using standardized conversion criteria [34,37,40]. Adolescent anxiety scores were calculated by aggregating responses to the GAD-7 [35]. Adolescent depression raw scores were calculated by aggregating responses to the PHQ-9A and then multiplied by 9, divided by 8, and rounded to the nearest whole number to account for the single omitted item. Inattention, hyperactivity, and oppositional symptom scores were calculated by aggregating the responses to the items in each of the symptom subsets of the SNAP-IV [41]. Symptom severity was determined for all child and adolescent symptoms using previously defined criteria [34,37,39,41-43].

For all analyses, caregiver burnout was reported based on response to the single item, and caregiver absenteeism was reported as severity of absenteeism and number of days of work missed. We chose this analytic approach to ensure that results are robust to minor changes to the screening and assessment methods (Multimedia Appendix 1). Therefore, for all outcomes except caregiver burnout and workplace absenteeism, symptom severity was analyzed as “elevated” or “not elevated.” For all symptoms except caregiver burnout, absenteeism, and parental stress, outcomes were classified as elevated if symptom severity was moderate to severe, and not elevated if the assessment was screened out or symptom severity was low to mild. A decrease in burnout or absenteeism from baseline (first assessment) was defined as a decrease in burnout score or a decrease in number of days of work missed, respectively.

### Statistical Analysis

#### Caregiver Burnout and Workplace Absenteeism at Baseline

Caregiver outcomes at baseline (first assessment before beginning care with the DMHI) were analyzed for all caregivers with complete caregiver assessments (n=6508). Caregivers with duplicate baseline assessments (n=2) were removed for a sample size of 6506. The following characteristics were described for these caregivers: mean child age (at baseline), child sex, child race or ethnicity, type of participation with the DMHI (BCM intake only, coaching only, coaching and therapy, or therapy only), and duration of participation with the DMHI (first event to last event). Burnout and absenteeism scores were reported, as well as group trends for the number of days of work missed. Cumulative link models (CLMs) were used to assess whether workplace outcomes were associated with caregiver’s comorbid symptoms. For burnout, the predictors were elevated absenteeism, elevated sleep problems, and elevated parental stress. For absenteeism, the predictors were elevated burnout, elevated sleep problems, and elevated parental stress. Child (child and adolescent, grouped for analyses) characteristics and mental health symptoms were assessed to identify associations between predictors and caregiver burnout and workplace absenteeism. Only caregivers of children and adolescents aged 6 years or older (given age validation for pediatric assessments) and whose child had all mental health assessments complete were included in analyses (n=5628; Multimedia Appendix 1). CLMs were used to determine whether the following child characteristics and mental health symptoms were associated with the severity of caregiver workplace outcomes (burnout and absenteeism): age (child vs teen), sex (female vs nonfemale), elevated depression, elevated anxiety, elevated inattention, elevated hyperactivity, elevated opposition, and elevated sleep problems.

#### Change in Caregiver Burnout and Workplace Absenteeism

Change in caregiver burnout and workplace absenteeism over their child’s care with the DMHI was assessed for caregivers with elevated burnout and elevated workplace absenteeism at baseline who also met the following inclusion criteria: baseline assessment completed within 2 months of beginning care with the DMHI (ie, for an accurate assessment of mental health status at care start), and had at least 1 completed follow-up assessment after the start of care with the DMHI (see Multimedia Appendix 1 for details on exclusions). Ultimately, 2121 were included in the analyses for change in burnout and 1327 were included in the analyses for change in absenteeism. Percentages of caregivers with a decrease and increase in workplace symptoms— for both burnout and absenteeism (number of days of work missed)—were reported for the caregivers’ first follow-up assessment during care as well as their last follow-up assessment during care. Percentages of caregivers with elevated workplace outcomes at these time points were also reported, and the percentages of caregivers with a decrease in workplace symptoms *reported at any time during care* were reported. Finally, percentages of caregivers with nonelevated workplace symptoms at baseline and elevated workplace symptoms at the last follow-up were reported. For absenteeism, reported decreases in the number of days worked were compared with zero using Wilcoxon signed rank tests.

For caregivers of a child with elevated mental health symptoms at baseline, the maximal percent decrease in child mental health symptom severity during care was calculated. The maximal decrease in child’s mental health symptom severity was compared between groups using Wilcoxon signed rank sums tests for caregivers with a decrease in workplace symptoms versus those with no decrease in workplace symptoms at last follow-up. Only caregivers of a child with elevated symptoms at baseline and a follow-up assessment after the start of care were included in these analyses (Multimedia Appendix 1; n=1739 included in burnout and n=1041 included in absenteeism).

CLMs were used to determine whether months in care were associated with lower severity of burnout and absenteeism, and comorbid caregiver symptoms, child characteristics, and child mental health symptoms were assessed as potential covariates in these models. The basic model for each workplace symptom included months in care as a fixed effect. Likelihood ratio tests were used to compare this basic model with an identical model including a single potential covariate added as a fixed effect. The potential covariates assessed were elevated comorbid caregiver symptoms, child’s age group (child vs teen), child’s sex (female vs nonfemale), and child’s mental health symptoms. Only predictors that significantly improved model fit were retained in the final model (see Multimedia Appendix 1 for comprehensive results from likelihood ratio tests). For burnout, all comorbid caregiver symptoms were retained, as well as child sex, and all child mental health symptoms. For absenteeism, all comorbid caregiver symptoms were retained, as well as the following child mental health symptoms: depression, anxiety, inattention, hyperactivity, and sleep. Only assessments taken after beginning care with the DMHI were considered in the CLMs.

For all CLMs, coefficient estimates were used to determine whether each predictor was associated with the severity of workplace symptoms. Throughout, the alpha-level was set to .05 for all analyses. Standard descriptive statistics (percentages; mean, SD; and median, IQR were used to describe data. Data were analyzed with RStudio (version 2023.03.0+386; Posit, PBC) [44].

## Results

### Overview

Overall, caregivers included in the analyses (N=6506) cared for children with a mean age of 10.58 (SD 3.81) years, and 49.86% (3244) of the children were female. In terms of reported race and ethnicity, 50.63% (3294) were white and 32.02% (2083) identified as “Other” or multiple race and ethnicity options. While all children had at least 1 session with a Bend Health practitioner, 16.25% (1057) completed only their BCM intake session, 59.67% (3882) were in coaching only, 22.69% (1476) were in coaching and therapy, and 1.40% (91) were in therapy only. The duration of participation in care with the DMHI was a median of 3.46 (1.7‐5.9) months.

### Caregiver Burnout and Workplace Absenteeism at Baseline

At baseline, 8.84% of caregivers (575) reported no symptoms of burnout, 45.20% (2941) reported low burnout, 33.60% (2186) reported mild burnout, 6.87% (447) reported moderate burnout, and 5.49% (357) reported severe burnout. In terms of absenteeism, 71.04% (4622) reported no missed work, 17.05% (1109) missed less than 1 week of work, 7.49% (487) missed 1-2 weeks of work, 2.29% (149) missed 2-3 weeks of work, and 2.14% (139) missed 3 or greater weeks of work. For those with any absenteeism (n=1884), they reported a median of 4 (2-7) days missed.

In terms of percentages of caregivers with elevated outcomes at baseline, 45.96% (2990) had elevated burnout, 28.96% (1884) had elevated absenteeism, 12.76% (830) had elevated sleep problems, and 17.57% (1143) had elevated parental stress. More severe levels of burnout were significantly associated with elevated absenteeism (*z*=15.30; *P*<.001), elevated sleep problems (*z*=20.41; *P*<.001), and elevated parental stress (*z*=15.30; *P*<.001). This same pattern was observed for severity of absenteeism; greater absenteeism was associated with elevated caregiver outcome severity of all types (all *P*<.001; Table 1).

More severe symptoms of burnout were significantly associated with having a younger child (child vs adolescent; *z*=3.20; *P*=.001), as well as having a child with elevated symptoms of any type (all *P*<.01). Child sex was not associated with the severity of burnout symptoms. More severe absenteeism was associated with having a child with elevated symptoms of depression (*z*=3.33; *P*<.001), anxiety (*z*=3.96; *P*<.001), inattention (*z*=2.48; *P*=.01), and hyperactivity (*z*=2.12; *P*=.03). Child age and sex, as well as opposition and sleep problems, were not associated with the severity of absenteeism. Comprehensive results for all predictors in these analyses are reported in Table 2.

**Table 1.** Results from analyses assessing whether the severity of caregiver symptoms is associated with elevated comorbid caregiver symptoms.

Outcome (caregiver outcome severity)	Predictor (elevated comorbid caregiver symptom)	Estimate (SE)	*z* value	*P* value
Burnout	Absenteeism	0.81 (0.05)	15.30	<.001
	Sleep problems	1.49 (0.07)	20.41	<.001
	Parental stress	1.44 (0.06)	20.39	<.001
Absenteeism	Burnout	0.83 (0.06)	13.96	<.001
	Sleep problems	0.56 (0.08)	7.24	<.001
	Parental stress	0.35 (0.07)	5.07	<.001

**Table 2.** Results from analyses assessing whether the severity of caregiver symptoms is associated with their child’s characteristics (demographics and symptoms).

Outcome (caregiver symptom severity)	Predictor (child’s characteristic)	Estimate (SE)	*z* value	*P* value
Burnout	Age (child aged 6-12 years)	0.18 (0.06)	3.20	.001
	Sex (female)	−0.03 (0.05)	−0.59	.56
	Depression (elevated)	0.19 (0.06)	3.03	.002
	Anxiety (elevated)	0.27 (0.06)	4.81	<.001
	Inattention (elevated)	0.43 (0.06)	7.01	<.001
	Hyperactivity (elevated)	0.26 (0.08)	3.23	.001
	Opposition (elevated)	0.46 (0.06)	7.23	<.001
	Sleep problems (elevated)	0.25 (0.05)	4.58	<.001
Absenteeism	Age (child aged 6-12 years)	0.07 (0.06)	1.11	.27
	Sex (female)	0.02 (0.06)	0.34	.74
	Depression (elevated)	0.24 (0.07)	3.33	<.001
	Anxiety (elevated)	0.26 (0.07)	3.96	<.001
	Inattention (elevated)	0.18 (0.07)	2.48	.01
	Hyperactivity (elevated)	0.20 (0.09)	2.12	.03
	Opposition (elevated)	0.11 (0.07)	1.51	.13
	Sleep problems (elevated)	0.10 (0.06)	1.57	.12

### Change in Caregiver Burnout and Workplace Absenteeism

**Figure 1.**
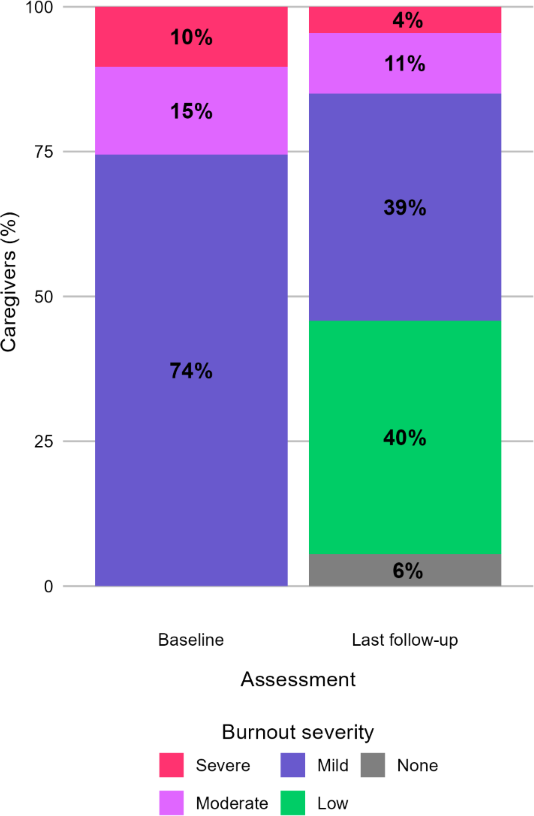
Burnout symptom severity for caregivers with elevated burnout at baseline and a follow-up assessment (n=2121) reported for the baseline and last follow-up assessments.

For 2121 caregivers with elevated burnout at baseline and a follow-up assessment after beginning care, the first-follow-up was completed after a median of 0.90 months (0.66‐1.13) in care with the DMHI. At this time, 49.88% (1058/2121) of caregivers with elevated burnout at baseline had a decrease in burnout symptom severity, 7.97% (169/2121) had an increase in severity, and 62.47% (1325/2121) still had elevated burnout. At the last follow-up assessment during care with the DMHI, taken after a median of 2.80 months (1.27‐5.40) in care, 56.58% (1200/2121) of caregivers with elevated burnout at baseline had a decrease in burnout symptom severity, 6.74% (143/2121) had an increase in severity, and 54.17% (1149/2121) still had elevated burnout. The percentages of burnout severity at baseline and last follow-up are reported in Figure 1. Overall, 68.64% (1456/2121) of caregivers reported a decrease in burnout at any point during care, with the first decrease reported after a median of 1.10 months (0.80‐1.97). At the last follow-up, children of caregivers with a decrease in burnout (n=977) had larger improvements in their own mental health than children of caregivers with no decrease in burnout (n=762; median −65.63%, IQR −100.00 to −40.00 vs median −52.51%, IQR −95.81 to −28.57; *z*=−5.22; *P*<.001). For caregivers with nonelevated burnout at baseline, 9.61% (338/3178) met the criteria for elevated burnout at their last follow-up during care.

In the analysis of burnout symptom severity during care with the DMHI, greater months in care was significantly associated with less severe symptoms of burnout (*z*=−5.76; *P*<.001). All elevated comorbid caregiver symptoms were associated with more severe burnout during care (all *P*<.001). The following child characteristics were associated with more severe symptoms of burnout: female sex (*z*=4.50; *P*<.001), elevated depression (*z*=2.01; *P*=.04), elevated hyperactivity (*z*=3.05; *P*=.002), and elevated sleep problems (*z*=4.18; *P*<.001). Child anxiety, inattention, and opposition were not associated with caregiver burnout during care (both *P*>.10).

**Figure 2.**
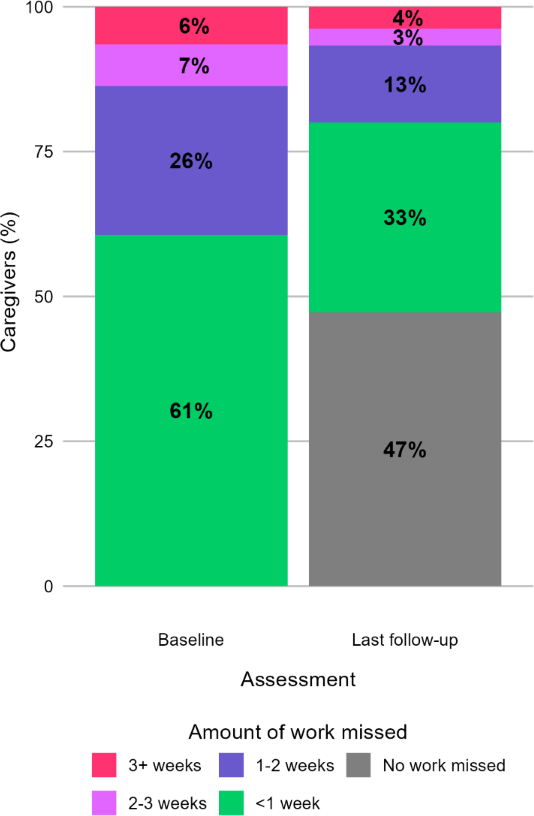
Amount of work missed for caregivers with elevated absenteeism at baseline and a follow-up assessment (n=1327) reported for the baseline and last follow-up assessments.

At the first follow-up assessment during care with the DMHI, taken after a median of 0.93 months (0.70‐1.20) in care, 73.25% (972/1327) of caregivers with elevated absenteeism at baseline had a decrease in number of days of work missed, 22.08% (293/1327) had an increase in days of work missed, and 60.14% (798/1327) still had elevated absenteeism. At the last follow-up assessment during care with the DMHI, taken after a median of 2.97 months (1.37‐5.45) in care, 76.56% (1016/1327) of caregivers with elevated absenteeism at baseline had a decrease in absenteeism, 19.22% (255/1327) had an increase in absenteeism, and 52.75% (700/1327) still had elevated absenteeism. The percentages of amount of work missed at baseline and last follow-up are reported in Figure 2. Overall, 87.26% (1158/1327) of caregivers reported a decrease in absenteeism at any point during care, with the first decrease reported after a median of 1.00 months (0.76‐1.57). Caregivers with elevated absenteeism at baseline had a median decrease of 2 (4-0) days of work missed at first follow-up (*z*=−17.9; *P*<.001), and 2 (4.5-.5) fewer days of work at last follow-up (*z*=−19.2; *P*<.001). At the last follow-up, children of caregivers with a decrease in absenteeism (n=802) had larger improvements in their own mental health than children of caregivers with no decrease in absenteeism (n=239; median −64.29%, IQR −100.00 to −39.13 vs median −57.14%, IQR −91.75 to −28.57; *z*=−2.65; *P*=.008). For caregivers with nonelevated absenteeism at baseline, 10.95% (506/4622) reported missing any amount of work at their last follow-up during care.

In the analysis of change in absenteeism over time, greater months in care was significantly associated with lower levels of absenteeism (*z*=−6.72; *P*<.001). Elevated comorbid burnout significantly associated with higher levels of absenteeism during care (*z*=6.89; *P*<.001) and elevated child anxiety was marginally associated with higher levels of absenteeism (*z*=1.75; *P*=.08). Elevated comorbid sleep and elevated comorbid stress, as well as having a child with elevated depression, inattention, hyperactivity, and sleep, were not associated with absenteeism during care.

## Discussion

### Principal Results

Using retrospective analysis, the purpose of this study was to (1) explore associations between caregiver burnout, absenteeism, comorbid symptoms, and child mental health problems among caregivers seeking mental health treatment for their children, and (2) assess whether caregiver burnout and absenteeism improved while their child participates in a pediatric DMHI.

Before beginning care with the DMHI, 46% (2990/6506) of caregivers had elevated burnout and 29% (1884/5606) had elevated workplace absenteeism. Child characteristics and mental health symptom presentation were significantly associated with their caregiver’s workplace symptoms. Furthermore, while their child participated in care, 69% (1456/2121) of caregivers reported a decrease in burnout and 87% (1158/1327) reported a decrease in absenteeism. These changes were notable after only 1 month in care, with 50% (1058/2121) of caregivers reporting reduced burnout, and nearly 3 in 4 (972/1327, 73%) reporting a reduction in the number of days of work missed. Greater time in the DMHI significantly associated with less severe workplace symptoms, and children of caregivers with improvement in workplace symptoms reported larger improvements in pediatric outcomes than those whose caregiver did have improvement in workplace symptoms.

We found that symptoms of elevated burnout and workplace absenteeism were prevalent in caregivers before starting care with the pediatric DMHI, as almost half and about one-third reported elevated burnout and absenteeism, respectively. It is well known that workplace burnout and absenteeism, which are at historic highs [45,46], are perpetuated by family-related stress, particularly a child’s mental health difficulties [14,47-51]. Parents spend a significant amount of time managing their children’s mental and behavioral health needs, which is exacerbated by lack of access to and knowledge of evidence-based mental health treatment options for their children [48,52]. Thus, it is unsurprising that percentages of burnout (2990/6506, 46%) and workplace absenteeism (1884/6506, 29%) were high among caregivers actively seeking treatment for their child’s mental health challenges.

Burnout was higher among those caring for a child with any elevated mental health symptom severity, and absenteeism was higher among those caring for a child with elevated internalizing (ie, anxiety and depression), inattention, and hyperactivity symptoms. These findings align with previous literature suggesting a reciprocal relationship, where a child’s mental health challenges exacerbate caregiver sleep problems and stress [53,54], which can contribute to caregiver burnout and absenteeism [55-57]. In turn, while some studies highlight the additional burden of parenting a child with mental health and behavioral challenges [6-9,11,58], caregiver burnout may exacerbate child mental health challenges by reducing the caregiver’s capacity to provide emotional support and consistent care [59,60]. Given the complex bidirectional relationship between child-caregiver well-being [54], it is critical to highlight interventions that may produce benefit for both caregivers and their children. As the first study to examine both caregiver absenteeism and burnout across multiple child mental health challenges, this work builds on prior studies that have focused on single conditions (eg, ADHD and autism) or isolated workplace outcomes [15], contributing to a broader understanding of the link between caregiver and child well-being.

While children were in care with the DMHI, their caregivers exhibited significant improvements in workplace symptoms. Burnout decreased in 69% (1456/2121) of caregivers and absenteeism decreased in 87% (1158/1327) at any point during care, with caregivers reporting that they missed 2 fewer days of work at the end of their child’s care than initially reported at baseline. Other studies have reported that caregivers whose children engage in traditional modes of mental and behavioral health care evince corresponding improvements, such as decreases in parental stress, sleep problems, anxiety, and depression [61,62]. In a study of caregivers and youth participating in a DMHI, Grodberg et al [48] found that increases in caregiver productivity throughout care were linked to increases in caregivers’ feelings of connectedness to their children. Similarly, our recent evidence also suggests that caregivers whose children engage in a pediatric DMHI report decreased parental stress and sleep problems [10]. The present findings extend this work by demonstrating that these caregiver benefits also translate into occupational outcomes, with greater time in care associated with reductions in both burnout and absenteeism. Notably, improvements in caregiver workplace symptoms were associated with larger improvements in child mental health symptoms during care. These results are largely in-line with the broader literature that indicates a bidirectional relationship between caregiver and child well-being [53-57]. Our findings highlight the opportunity for employers to manage employees’ burnout and absenteeism by providing mental health care not only for employees themselves but also for their children and families.

### Strengths and Limitations

Our study has several strengths. This is the first study, to our knowledge, to address the potential downstream benefits of pediatric care with a DMHI on the workplace symptoms of their caregivers. The deterioration of parent and caregiver well-being has been increasing in prevalence and was called out in 2024 by the US Surgeon General as a critical problem [4]. We demonstrate that addressing child mental health may be an effective avenue to reduce caregiver strain and workplace problems. From an analytic standpoint, this study draws data from a large sample, with more than 6000 caregivers included in analyses of mental health symptoms at baseline, more than 2000 change in burnout analyses, and more than 1000 for change in absenteeism analyses. Additionally, this study assesses outcomes associated with an established, commercial, and successful DMHI, which has been shown to be effective in addressing child and adolescent mental health challenges [18,19,25]. Our findings overall contribute to a growing body of evidence supporting the use of DMHIs—which are typically more flexible and accessible than traditional care—to address both pediatric and caregiver well-being.

There are some limitations of this study. This was a retrospective study that prevented us from drawing any causal conclusions, and we relied on self-report measures to obtain information regarding all outcomes of interest. Further research using an experimental design and using objective measures where possible (eg, reports of caregiver absences from work) is necessary to determine whether involvement in a pediatric DMHI precipitates improvements in caregiver workplace symptoms. While some improvement may be expected due to regression to the mean following a child’s referral to care, our findings suggest that greater time in care contributes to continued reductions in burnout and absenteeism. Controlled studies are needed to further clarify the extent to which these changes reflect treatment effects versus natural symptom resolution over time. We also did not collect demographic information on caregivers, which limits our ability to examine whether caregiver characteristics (eg, age and biological sex) influenced burnout and absenteeism.

Another limitation of this study is that we did not ask caregivers whether they were employed, their type of employment, or their hours worked per week. The burnout and absenteeism measures used in this study do not define “work,” and caregivers responded based on their interpretation of what “work” was, full-time, part-time, paid, or unpaid. As a result, we were unable to differentiate between burnout and absenteeism among caregivers who were employed in different capacities or engaged in informal caregiving roles. Without these data, we cannot determine whether reductions in absenteeism reflect actual changes in workplace behavior, time away from caregiving responsibilities, or broader improvements in caregiver functioning. Future studies should consider frequency of occurrence and types of employment and inclusion of caregivers who engage in informal caregiving and other unpaid work when considering burnout and absenteeism [63]. We used a single-item burnout measure and a single section from the HPQ questionnaire to assess absenteeism, which prevented us from comprehensively assessing our workplace outcomes. These decisions were made to simplify the assessment process and limit burden. While we considered the possibility that improvements in child mental health could be linked to improvements in caregiver workplace symptoms, we did not examine the specific types of mental health symptoms or care targets, as these were beyond the scope of this study. Future studies should account for a variety of children’s mental health symptoms and more comprehensively assess caregivers’ burnout, absenteeism, and presenteeism to address these limitations.

Finally, the nature of administering assessments and screeners changed partway through the study. Given that the burnout assessment did not have a corresponding screener and the absenteeism screener was simply used to identify no absenteeism, we do not expect that these methodological changes had any substantive impact on our main findings. While the total scores for the other caregiver and child mental health outcomes may have been affected by the changes in the screening methods, we only assessed whether elevated versus nonelevated symptoms predicted caregiver workplace outcomes. Thus, we expect that our findings are robust to these small methodological changes.

### Conclusions

Caregivers are growing increasingly concerned about their children’s mental health, which in turn is impacting their well-being and performance in the workplace. This study provides promising preliminary evidence that caregivers show decreases in their burnout and absenteeism when their children participate in a pediatric DMHI. As such, employers should consider offering pediatric digital mental health care to employees with children experiencing mental health difficulties, which may mitigate burnout and absenteeism. While these findings highlight the potential benefits of digital mental health care for working caregivers, future research should compare DMHIs with traditional face-to-face mental health care to determine their relative effectiveness and accessibility.

## Supplementary material

10.2196/67149 Multimedia Appendix 1 Supplemental methods.

## Abbreviations

ADHD: attention-deficit/hyperactivity disorder
BCM: behavioral care manager
CLM: cumulative link model
DMHI: digital mental health intervention
*DSM-V-TR*: *Diagnostic and Statistical Manual of Mental Disorders* (Fifth Edition, Text Revision)
GAD-7: Generalized Anxiety Disorder-7
HPQ: Health and Work Performance Questionnaire
ISI: Insomnia Severity Index
PHQ-9A: Patient Health Questionnaire-9 adolescent version
PROMIS: Patient-Reported Outcomes Measurement Information System
PSS: Parental Stress Scale
SNAP-IV: Swanson Nolan and Pelham Rating Scale version 4

## References

[1] Henderson MD, Schmus CJ, McDonald CC, Irving SY (2020). The COVID-19 pandemic and the impact on child mental health: a socio-ecological perspective. Pediatr Nurs.

[2] Lebrun-Harris LA, Ghandour RM, Kogan MD, Warren MD (2022). Five-year trends in US children’s health and well-being, 2016-2020. JAMA Pediatr.

[3] Sloan CJ, Mailick MR, Hong J, Ha JH, Greenberg JS, Almeida DM (2020). Longitudinal changes in well-being of parents of individuals with developmental or mental health problems. Soc Sci Med.

[4] (2024). Parents under pressure: the US surgeon general’s advisory on the mental health & well-being of parents. US Surgeon General.

[5] Patrick SW, Henkhaus LE, Zickafoose JS (2020). Well-being of parents and children during the COVID-19 pandemic: a national Survey. Pediatrics.

[6] Lohaus A, Chodura S, Möller C (2017). Children’s mental health problems and their relation to parental stress in foster mothers and fathers. Child Adolesc Psychiatry Ment Health.

[7] Theule J (2010). Predicting Parenting Stress in Families of Children With ADHD.

[8] Alfano CA, Gamble AL (2009). The role of sleep in childhood psychiatric disorders. Child Youth Care Forum.

[9] Cortese S, Faraone SV, Konofal E, Lecendreux M (2009). Sleep in children with attention-deficit/hyperactivity disorder: meta-analysis of subjective and objective studies. J Am Acad Child Adolesc Psychiatry.

[10] Huffman LG, Lawrence-Sidebottom D, Huberty J (2023). Sleep problems and parental stress among caregivers of children and adolescents enrolled in a digital mental health intervention. Front Child Adolesc Psychiatry.

[11] Mesman J, Koot HM (2000). Child-reported depression and anxiety in preadolescence: I. Associations with parent- and teacher-reported problems. J Am Acad Child Adolesc Psychiatry.

[12] Bakker A, Demerouti E (2013). Job demands-resources model of burnout. J Work Organ Psychol.

[13] Christenson JD, Crane DR, Malloy J, Parker S (2016). The cost of oppositional defiant disorder and disruptive behavior: a review of the literature. J Child Fam Stud.

[14] Gawlik KS, Melnyk BM, Mu J, Tan A (2022). Psychometric properties of the new working parent burnout scale. J Pediatr Health Care.

[15] Noe L, Hankin C (2001). Pmh10: health outcomes of childhood attention-deficit/hyperactivity disorder (ADHD): health care use and work status of caregivers. Value Health.

[16] Schonfeld IS, Bianchi R (2021). From Burnout to Occupational Depression: Recent Developments in Research on Job-Related Distress and Occupational Health. Front Public Health.

[17] Strömberg C, Aboagye E, Hagberg J, Bergström G, Lohela-Karlsson M (2017). Estimating the effect and economic impact of absenteeism, presenteeism, and work environment-related problems on reductions in productivity from a managerial perspective. Value Health.

[18] Huffman LG, Lawrence-Sidebottom D, Huberty J (2023). Using digital measurement-based care for the treatment of anxiety and depression in children and adolescents: observational retrospective analysis of Bend Health data. JMIR Pediatr Parent.

[19] Lawrence-Sidebottom D, Huffman LG, Huberty J (2023). Using digital measurement-based care to address symptoms of inattention, hyperactivity, and opposition in youth: retrospective analysis of Bend Health. JMIR Form Res.

[20] Bryant BR, Sisk MR, McGuire JF (2024). Efficacy of gamified digital mental health interventions for pediatric mental health conditions: a systematic review and meta-analysis. JAMA Pediatr.

[21] Khanna MS, Carper M (2022). Digital mental health interventions for child and adolescent anxiety. Cogn Behav Pract.

[22] Alvarez-Jimenez M, Gleeson JF, Rice S, Gonzalez-Blanch C, Bendall S (2016). Online peer-to-peer support in youth mental health: seizing the opportunity. Epidemiol Psychiatr Sci.

[23] Catarino A, Harper S, Malcolm R (2023). Economic evaluation of 27,540 patients with mood and anxiety disorders and the importance of waiting time and clinical effectiveness in mental healthcare. Nat Mental Health.

[24] Jankovic D, Bojke L, Marshall D (2021). Systematic review and critique of methods for economic evaluation of digital mental health interventions. Appl Health Econ Health Policy.

[25] Lawrence-Sidebottom D, Huffman LG, Beam A (2024). Improvements in sleep problems and their associations with mental health symptoms: a study of children and adolescents participating in a digital mental health intervention. Digit Health.

[26] Rohland BM, Kruse GR, Rohrer JE (2004). Validation of a single‐item measure of burnout against the Maslach burnout inventory among physicians. Stress Health.

[27] Dolan ED, Mohr D, Lempa M (2015). Using a single item to measure burnout in primary care staff: a psychometric evaluation. J Gen Intern Med.

[28] Mateo-Rodríguez I, Knox E, Oliver-Hernandez C, Daponte-Codina A (2023). Validation of a single-item screening measure of burnout in a sample of Spanish health workers. Soc Sci (Basel).

[29] West CP, Dyrbye LN, Satele DV, Sloan JA, Shanafelt TD (2012). Concurrent validity of single-item measures of emotional exhaustion and depersonalization in burnout assessment. J Gen Intern Med.

[30] Kessler RC, Barber C, Beck A (2003). The World Health Organization health and work performance questionnaire (HPQ). J Occup Environ Med.

[31] Bastien CH, Vallières A, Morin CM (2001). Validation of the insomnia severity index as an outcome measure for insomnia research. Sleep Med.

[32] Berry JO, Jones WH (1995). The Parental Stress Scale: initial psychometric evidence. J Soc Pers Relat.

[33] American Psychiatric Association (2013). Diagnostic and Statistical Manual of Mental Disorders.

[34] Irwin DE, Gross HE, Stucky BD (2012). Development of six PROMIS pediatrics proxy-report item banks. Health Qual Life Outcomes.

[35] Mossman SA, Luft MJ, Schroeder HK (2017). The generalized anxiety disorder 7-item scale in adolescents with generalized anxiety disorder: signal detection and validation. Ann Clin Psychiatry.

[36] Kroenke K, Spitzer RL, Williams JB (2001). The PHQ-9: validity of a brief depression severity measure. J Gen Intern Med.

[37] Forrest CB, Meltzer LJ, Marcus CL (2018). Development and validation of the PROMIS pediatric sleep disturbance and sleep-related impairment item banks. Sleep.

[38] Kumari R, Kohli A, Malhotra P, Grover S, Khadwal A (2018). Burden of caregiving and its impact in the patients of acute lymphoblastic leukemia. Ind Psychiatry J.

[39] Singh NN, Lancioni GE, Winton ASW (2014). Mindfulness-based positive behavior support (MBPBS) for mothers of adolescents with autism spectrum disorder: effects on adolescents’ behavior and parental stress. Mindfulness (N Y).

[40] Sherlock P, Blackwell CK, Kallen MA (2022). Measuring PROMIS® emotional distress in early childhood. J Pediatr Psychol.

[41] Swanson JM, Sandman CA, Deutsch C, Baren M (1983). Methylphenidate hydrochloride given with or before breakfast: I. Behavioral, cognitive, and electrophysiologic effects. Pediatrics.

[42] Hall CL, Guo B, Valentine AZ (2020). The validity of the SNAP-IV in children displaying ADHD symptoms. Assessment.

[43] Johnson JG, Harris ES, Spitzer RL, Williams JBW (2002). The patient health questionnaire for adolescents: validation of an instrument for the assessment of mental disorders among adolescent primary care patients. J Adolesc Health.

[44] RStudio Team RStudio: Integrated Development for R.

[45] (2022). Labor force statistics from the current population survey. US Bureau of Labor Statistics.

[46] Abramson A (2022). Burnout and stress are everywhere. Monit Psychol.

[47] Ford MT, Heinen BA, Langkamer KL (2007). Work and family satisfaction and conflict: a meta-analysis of cross-domain relations. J Appl Psychol.

[48] Grodberg D, Bridgewater J, Loo T, Bravata D (2022). Examining the relationship between pediatric behavioral health and parent productivity through a parent-reported survey in the time of COVID-19: exploratory study. JMIR Form Res.

[49] Krouse SS, Afifi TD (2007). Family-to-work spillover stress: coping communicatively in the workplace. J Fam Commun.

[50] Roskam I, Aguiar J, Akgun E (2021). Parental burnout around the globe: a 42-country study. Affect Sci.

[51] Stracke M, Heinzl M, Müller AD (2023). Mental health is a family affair-systematic review and meta-analysis on the associations between mental health problems in parents and children during the COVID-19 pandemic. Int J Environ Res Public Health.

[52] Coe E, Enomoto K, Herbig B, Kothari A, Steuland J (2021). COVID-19 and burnout are straining the mental health of employed parents.

[53] Martin CA, Papadopoulos N, Chellew T, Rinehart NJ, Sciberras E (2019). Associations between parenting stress, parent mental health and child sleep problems for children with ADHD and ASD: systematic review. Res Dev Disabil.

[54] Stone LL, Mares SHW, Otten R, Engels R, Janssens JMAM (2016). The co-development of parenting stress and childhood internalizing and externalizing problems. J Psychopathol Behav Assess.

[55] Bailey SK, Haggarty J, Kelly S (2016). Global absenteeism and presenteeism in mental health patients referred through primary care. WOR.

[56] Bubonya M, Cobb-Clark DA, Wooden M (2017). Mental health and productivity at work: does what you do matter?. Labour Econ.

[57] Hilton MF, Sheridan J, Cleary CM, Whiteford HA (2009). Employee absenteeism measures reflecting current work practices may be instrumental in a re-evaluation of the relationship between psychological distress/mental health and absenteeism. Int J Methods Psychiatr Res.

[58] Becker SP (2020). ADHD and sleep: recent advances and future directions. Curr Opin Psychol.

[59] Meltzer H, Ford T, Goodman R, Vostanis P (2011). The burden of caring for children with emotional or conduct disorders. Int J Family Med.

[60] Mikolajczak M, Raes ME, Avalosse H, Roskam I (2018). Exhausted parents: sociodemographic, child-related, parent-related, parenting and family-functioning correlates of parental burnout. J Child Fam Stud.

[61] Katzmann J, Döpfner M, Görtz-Dorten A (2018). Child-based treatment of oppositional defiant disorder: mediating effects on parental depression, anxiety and stress. Eur Child Adolesc Psychiatry.

[62] Mihaila I, Hartley SL (2018). Parental sleep quality and behavior problems of children with autism. Autism.

[63] Gérain P, Zech E (2019). Informal caregiver burnout? Development of a theoretical framework to understand the impact of caregiving. Front Psychol.

