# Supplementary material for: Evaluating the Impact of Pediatric Digital Mental Health Care on Caregiver Burnout and Absenteeism: Longitudinal Observational Study
Source: JMIR Pediatr Parent. 2025 Jun 27;8:e67149. doi: 10.2196/67149 (PMC12229275; doi:10.2196/67149)
Supplement: Multimedia Appendix 1 [file pediatrics-v8-e67149-s001.docx]

Supplemental material

# Methods

## Measures

### Child mental health symptoms

To flag children ages one to 12 with anxiety or depressive symptoms, caregivers of children ages one to 12 (caregiver-report) reported on the frequency of their child’s symptoms *over the last two weeks* by responding to the anxiety and depression screener questions from the DSM-5-TR Cross-Cutting Symptom Measure [1]. There are two anxiety symptom screener questions and three depressive symptom screener questions, each including a statement about a symptom or behavior a child may have (e.g., “not been able to stop worrying”). Caregivers of child members select the best-fit response using a 5-item Likert scale (0 = Not at all, 4 = Nearly every day). If a caregiver’s response to any child anxiety or depressive symptom screener question is two or greater (“Several days” or more frequently), the caregiver is prompted to complete the corresponding PROMIS assessment (anxiety or depression) [2, 3]. The PROMIS anxiety measure includes 10 questions about a child’s feelings of worry, as well as anxiety-related behaviors. The PROMIS depression measure includes 11 questions about a child’s feelings of sadness and depressive-related behaviors. Responses to both PROMIS measures are made on a 5-item Likert-type scale (1 = Never, 5 = Almost always).

To flag children ages 13 to 17 with anxiety or depressive symptoms, children ages 13 to 17 respond (self-report) to screener questions derived from the Generalized Anxiety Disorder 2-item (GAD-2) and Patient Health Questionnaire 2-item (PHQ-2), respectively, which each consist of the first two questions from the Generalized Anxiety Disorder 7-item (GAD-7) and the Patient Health Questionnaire 9-item (Adolescent modified; PHQ-9A). The questions from these assessments probe the frequency of anxiety- and depression-related behaviors and thoughts over the past two weeks [1]. Responses to these items are made using a 4-item Likert scale (0 = Not at all, 3 = Nearly every day). If the sum of a response to the anxiety or depressive symptom screener questions is two or greater, the child is prompted to complete the remaining questions from the Generalized Anxiety Disorder 7-item (GAD-7) or the Patient Health Questionnaire 9-item (Adolescent modified; PHQ-9A). The GAD-7 includes 7 questions about feelings of worry, fear, and stress, as well as difficulty relaxing or regulating irritation [4]. The original PHQ-9A includes 9 questions about feelings of sadness and frequency of depression-related behaviors [5]. The PHQ-9A used in this study omitted the item about suicidal ideation. No items were omitted from the GAD-7, and thus the anxiety symptom assessment included up to 7 items and the depressive symptom assessment included 8 questions.

To flag children ages one to 17 with inattention, hyperactivity, or oppositional symptoms, the caregiver responds to the two DSM-5-TR Cross-Cutting Symptom Measure questions for inattention/hyperactivity symptoms (one question for both) and oppositional symptoms (one question) [1]. Responses are made on a 5-item Likert-type scale (0 = Not at all, 4 = Nearly every day). If the response to the inattention/hyperactivity screener question is one or greater, the caregiver is prompted to complete the first 18 items of SNAP-IV measure [6]. If the response to the oppositional symptom screener question is one or greater, the caregiver is prompted to complete items 19 to 26 of the SNAP-IV measure. The full SNAP-IV includes 26 items about behaviors related to inattentiveness (questions 1 to 9), hyperactivity (questions 10 to 18), and oppositional/defiant behaviors (questions 19 to 26). Responses are made on a 4-item Likert-type scale (0 = Not at all, 3 = Very much).

To flag children ages one to 12 with sleep problems, caregivers (caregiver-report) respond to a single screener question: “During the past two (2) weeks, how much (or how often) has your child had problems sleeping--that is, trouble falling asleep, staying asleep, or waking up too early?” Children ages 13 to 17 (self-report) are asked to respond to the following screener question: “During the past two (2) weeks, how much (or how often) have you been bothered by not being able to fall asleep or stay asleep, or by waking up too early?” Best-fit responses to both questions are made on a 5-item Likert-type scale (0 = Not at all, 4 = Nearly every day). If the response to the screener question is two or greater (several days or more frequent), caregivers of children ages one to 12 (caregiver-report) and children ages 13 to 17 (self-report) complete the short form of the PROMIS sleep measure [7], which includes 7 items about sleep quality and satisfaction with sleep. Best-fit responses to the items are selected on the following 5-item Likert-type scales: Not at all to very much (items 1 to 6) and very poor to very good (item 7). All questions in the caregiver-report version of the sleep measure are directed to the caregiver, for example: “Their sleep was restless” and “They had trouble staying asleep.” Whereas questions for the self-report version are directed to the child themselves, for example: “My sleep was restless” and “I had trouble staying asleep.” Otherwise, the two versions (caregiver-report and self-report) were identical.

## Statistical analysis

In the analyses of child characteristics as predictors of caregiver outcomes, n=693 caregivers were excluded for child age (ages < 6 years not retained) and an additional n=185 were excluded for incomplete child assessments at baseline. Thus, these analyses included n=5,628 caregivers.

For the analyses of change in caregiver symptoms, the exclusions are as follows. For the burnout analyses, n=3516 caregivers were excluded because they did not have elevated burnout at baseline, n=57 were excluded due to timing of the baseline assessment, and n=812 were excluded because they did not have any follow-up assessments. For the workplace absenteeism analyses, n=4,622 caregivers were excluded because they did not have elevated absenteeism at baseline, n=43 were excluded due to timing of the baseline assessment, and n=514 were excluded because they did not have any follow-up assessments. Thus, the main analyses included n=2,121 caregivers for burnout and 1,327 for absenteeism.

For the comparison between-groups of child’s percent improvement in symptoms, an additional n=382 were excluded from burnout and an additional n=286 from absenteeism because the child did not have elevated symptoms at baseline or due to missingness of follow-up assessments (n=1,739 included in burnout and n=1,041 included in absenteeism). For the cumulative link analyses (CLM) of symptom severity over time, n=38 were excluded from burnout and n=3 from absenteeism for missingness of baseline covariate information (n=2,083 included in burnout and n=1,324 included in absenteeism).

The results from the likelihood ratio tests (LRTs) for the CLMs of burnout scores over time are as follows: caregiver absenteeism (*P*<.001), caregiver sleep problems (*P*<.001), caregiver stress (*P*<.001), child age group (*P*=.13), child sex (*P*<.001), child depression (*P*<.001), child anxiety (*P*<.001), child inattention (*P*<.001), child hyperactivity (*P*<.001), child opposition (*P*<.001), and child sleep (*P*<.001). The results from the LRTs for the CLMs of absenteeism over time are as follows: caregiver burnout (*P*<.001), caregiver sleep problems (*P*<.001), caregiver stress (*P*=.002), child age group (*P*=.33), child sex (*P*=.43), child depression (*P*=.002), child anxiety (*P*<.001), child inattention (*P*=.004), child hyperactivity (*P*=.006), and child opposition (*P*=.14), and child sleep (*P*=.006).

**References**

1. American Psychiatric Association. Diagnostic and statistical manual of mental disorders: Dsm-5. Washington, D.C.: American Psychiatric Association; 2013.

2. Irwin D.E., Gross H.E., Stucky B.D., Thissen D., DeWitt E.M., Lai J.S., et al. Development of six promis pediatrics proxy-report item banks. *Health Qual Life Outcomes* **2012**,*10*,22.

3. Sherlock P., Blackwell C.K., Kallen M.A., Lai J.S., Cella D., Krogh-Jespersen S., et al. Measuring promis(r) emotional distress in early childhood. *J Pediatr Psychol* **2022**,*47*(5),547-58.

4. Spitzer R.L., Kroenke K., Williams J.B., Lowe B. A brief measure for assessing generalized anxiety disorder: The gad-7. *Arch Intern Med* **2006**,*166*(10),1092-7.

5. Johnson J.G., Harris E.S., Spitzer R.L., Williams J.B. The patient health questionnaire for adolescents: Validation of an instrument for the assessment of mental disorders among adolescent primary care patients. *J Adolesc Health* **2002**,*30*(3),196-204.

6. Swanson J.M., Sandman C.A., Deutsch C., Baren M. Methylphenidate hydrochloride given with or before breakfast: I. Behavioral, cognitive, and electrophysiologic effects. *Pediatrics* **1983**,*72*(1),49-55.

7. Bevans K.B., Gardner W., Pajer K.A., Becker B., Carle A., Tucker C.A., et al. Psychometric evaluation of the promis(r) pediatric psychological and physical stress experiences measures. *J Pediatr Psychol* **2018**,*43*(6),678-92.
